# Impact of comorbidity on adverse drug reaction profile in a cohort of patients treated with Artemisinin combination therapies for uncomplicated malaria in Nigeria

**DOI:** 10.1002/prp2.302

**Published:** 2017-03-24

**Authors:** Peter U. Bassi, Adeline I. Osakwe, Comfort K. Ogar, Cassandra Elagbaje, Biyaya B. Nwankwo, Sulayman T. Balogun, Godwin N. Ntadom, Ambrose O. Isah

**Affiliations:** ^1^ Department of Pharmacology & TherapeuticsCollege of Health SciencesUniversity of Abuja FCTAbujaNigeria; ^2^ National Pharmacovigilance centreNAFDAC NigeriaAbujaNigeria; ^3^ Department of Community MedicineCollege of Health SciencesUniversity of Abuja FCTAbujaNigeria; ^4^ Department of Clinical Pharmacology & TherapeuticsCollege of Medical SciencesUniversity of MaiduguriBorno StateNigeria; ^5^ National Malaria control ProgrammeFMoHAbujaNigeria; ^6^ University of Benin Teaching HospitalBenin CityNigeria

**Keywords:** Comorbidity, ACTs, ADRs, Uncomplicated Malaria, Nigeria

## Abstract

Artemisinin‐based combination antimalarial therapy (ACTs), is still highly effective in uncomplicated falciparum malaria, however, there remain some concerns in relation to its safety and tolerability. Comorbid disease conditions may influence susceptibility to adverse drug reactions (ADRs) as the presence of multiple disease conditions may predisposes patients to ADRs due to the use of many medicines. There is therefore need to assess the impact of comorbidities on the ADR profile of malaria patients treated with ACTs. The study was carried out in health care facilities spread across Nigeria. From the database of over 10,000 patients recruited into an initial cohort, data for 1000 patients with comorbidities was extracted and matched with a control group of 1000 randomly selected patients with no comorbidity. There were 1105 adverse drug reactions in all, of which 66.2% were recorded in patients with comorbidity, and 34% are patients without comorbidity. The mean age of patients with comorbidities was 38.3 ± 17.5 years and 23.8 ± 17.2 for those without comorbidity. Out of the 979 patients with comorbidity, 36% were hypertensive, 2.2% hypertensive‐diabetes, 16.4% peptic ulcer disease, 10.4% HIV/AIDS, 4.4% diabetes and 4.3% were asthmatic. Patients with comorbidity were three times more likely to have adverse drug reaction than those without comorbidity (Odds ration = 2.96; 95% CI = 2.23–3.93). HIV/AIDS and osteoarthritis were significantly associated with development of adverse drug reactions. Probability was <0.0001. Age, weight, and height of patients were also found to be risk factor for development of adverse drug reactions.

**Abbreviations**
ABC: abacavir
ACT: artemisinin‐based combination therapy
ADR: adverse drug reactions
AL: artemether‐lumefantrine
SJS: Stevens–Johnson syndrome
TEN: toxic epidermal necrosis

## Introduction

Currently recommended combination antimalarial therapy for treatment of falciparum malaria, is artemisinin‐based combination therapies (ACTs). Artesunate + amodiaquine (AS + AQ) and artemether‐lumefantrine (AL), have been adopted by many countries in sub‐Saharan Africa, and Nigeria. These combinations therapy is still highly effective going by reports from clinical trials (FMoH Abuja 2011; Greenwood et al. 2005). However, the safety and tolerability of these drug combinations still pose some concern to healthcare providers and may be source of treatment interruption among the populations (Simooya et.al 2005), more so in the presence of comorbidities and use of other medicines these concern might be heightened. Recent reviews of studies of the safety of these new combination regimens has shown safety reassurance, identifying no serious concerns, but to date evaluations of drug safety and tolerability have been limited (Simooya et. al. 2005; Bassi et al. 2012; Fanello et al. 2006; Karema et al. 2006; Bukirwa et al. 2006; Kamya et al. 2007). Most of the ACTs safety data have been from clinical trials treatment evaluating single episodes of malaria in the absence of comorbidities.

Taking several medicines, whether prescription or over‐the‐counter, contributes to the risk of having an ADR. The number and severity of ADRs increases disproportionately as the number of drugs taken increases. “Many definitions are applied for polypharmacy. It is different from scholar to scholar but the basic concept of taking more medications at the same time than are clinically appropriate remains constant” (Rambhade et al. 2012). It implies to the prescription of too many medications for a particular patient, with a possibility of increased risk of ADRs. The more the number of medications that are prescribed the greater the possibility of polypharmacy, but this does not necessarily mean however that patients should not take many medications (Bushardt et al. 2008).

Drugs that are useful in the management of one disease may precipitate or worsen another, e.g., some beta‐blockers taken for heart disease or high blood pressure can worsen asthma and make it hard for people with diabetes to tell when their blood sugar is too low (Kurnik et al. 2004). In addition prednisone which is used for treating several conditions can aggravate congestive heart failure and cause fluid retention. Some of these interactions may have an insidious onset, and as a result careful and close medical attention is mandatory (Boer et al.^2003^). Concomitant patient's disease may also influence susceptibility to ADRs (Rambhade et al. 2012). For example, increase in the frequency of idiosyncratic toxicity with anti‐infective drugs such as trimethomprim‐sulphamethoxazole (Hanses et al. 2009). The presence of multiple disease conditions predispose patients to ADRs due to the combination of the presence of many diseases and the use of many medicines (Muaed 2012). AIDS for instance predisposes to ADRs, such that the incidence of Stevens–Johnson syndrome (SJS) and toxic epidermal necrosis (TEN) have been reported to be higher among those particular patients. A study showed that one of the most commonly used medications among patients who developed TEN and SJS is trimethoprim/sulfamethoxazole (Boer et al. 2003; Mittman et al. 2012; Muaed 2012). “A recent study of a multivariate logistic regression indicated that a T CD4 + count of lower than 200 cells/mm3 increased the risk of hepatotoxicity by a factor of 1.233(*P* < 0.001) and that coinfection with hepatitis B or C viruses increased this risk by a factor of 18.187” (*P* = 0.029) (Lima and Melo 2012). There is a paucity of publications on ADRs in patients with malaria and other comorbidities treated with ACTs. Therefore, this study seeks to assess the impact comorbidity on the ADR profile of in malaria patients taking ACTs. Specifically, the objectives of the study were to evaluate the incidence of adverse drug events among the cohort of patients with and without comorbidities, estimate the risk of developing adverse events among patients with and without comorbidities, identify the types and frequency of adverse drug reaction (or AEs) in patients with and without comorbidity and determine comorbidities that are associated with development of AEs.

## Subjects and Methods

The study was part of a larger observational, prospective cohort event monitoring program of adverse events in a cohort of patients treated with either Artemeter‐Lumefantrine (AL) or Artesunate Amodiaquine (AA), carried out in 18 health care facilities spread across the six geopolitical zones of Nigeria between 31st and May 2012.

From the database of the over 10,000 patients in the CEM study, we identified 1000 patients with comorbidity (all patients in the database with comorbidities) and extracted their demographic and clinical data from the database and compared with a group of 1000 patients from the same database systematically (every 10th patients ID was pick until we selected 1000 and if the 10 is with comorbidity, the next number is picked) selected patients who received ACTs but had no comorbidity.

All age groups and both sexes with the diagnosis of malaria with or without co‐morbidity on treatment with ACT and other drugs were included in the study group. The selected patients were categorized into group “A” those with comorbidity and group “B” those without comorbidity while study medications were assessed for adverse events beginning with the day 0 (first treatment day) of malaria.

An adverse event was defined as any untoward medical occurrence, irrespective of its suspected relationship to the study medications as per International Conference of Harmonization (ICH) guidelines (ICH‐E6 1996). A serious adverse event was defined as any adverse experience that resulted in death, life‐threatening experience, participant hospitalization, persistent or significant disability or incapacity, or specific medical or surgical intervention to prevent serious outcome.

Data of 2000 patients was analysed with for statistical analyses using SPSS VER 21. (IBM–SPSS 2011). Result was then presented as frequency distribution, percentages, and Chi Square to study relationships. Results were also presented in tables. Potential risk factors such as age, drug regimen, gender, pregnancy, and use of traditional medications to statistical were subjected to analysis using multinomial logistic regressions.

## Results

### Socio‐demographic characteristics

A total of 1979 patient suspected adverse events to ACT with or without comorbidity were reported. There were 979 patients with comorbid conditions, mean age 38.3 ± 17.5 years and 1000 patients without comorbidity 23.8 ± 17.2 years (range 3 months‐89 years), *P* < 0.001, with M/F sex ratio of 1.13 *P* < 0.001 that was studied (Table 1). The mean weight of the patients was 64.6 ± 22.7 kg and 50.1 ± 27.0 kg, *P* < 0.001 among those with comorbidity and without comorbidity, respectively (Table 2). 45.9% (913) patients received AA while 54.1% (1075) received AL among the cohort.

**Table 1:** Demographic characteristics

Age (year)	Comorbidity (*n* = 979)	No comorbidity (*n* = 999)	*χ* ^2^	*P*‐value
	Frequency (%)	Frequency (%)		
<4	55 (5.6)	188 (18.8)	79.960	<0.001^a^
4–8.9	44 (4.5)	103 (10.3)	24.311	<0.001^a^
9–14.9	27 (2.8)	70 (7.0)	19.143	<0.001^a^
≥15	853 (87.1)	638 (63.9)	144.214	<0.001^a^
Mean age	38.3 ± 17.5	23.8 ± 17.2		
Median age	40	25		
Sex				
Male	279 (28.5)	445 (44.5)	54.863	<0.001^a^
Female	700 (71.5)	554 (55.5)		

Student's *t* = 18.465; *P* < 0.001.

Mean difference = 14.4 (95% CI of difference = 12.9–16.0).

a Statistically significant *df* = 1.

### Symptoms and signs of Malaria

A total of 9189 signs and symptoms of suggestive of malaria were reported among 1988 patients, making an average 4–5 symptoms (Table 2) of malaria per patient at presentation. The most common symptoms were fever 1052 (11.5%), headache 1046(11.4%), weakness 958 (10.4%), joint pains 905(9.9%), tiredness 888 (9.2%), body pains 844 (9.2%), etc as seen in Table 3. All signs and symptoms were mild to moderate.

**Table 2:** Anthropometrics

Variable	Mean ± Standard deviation		Student's *t* statistic	*P*‐value	Mean diff	95% CI of diff	
	Comorbidity (*n* = 979)	No Comorbidity (*n* = 979)				Lower	Upper
Weight (kg)	64.6 ± 22.7	50.1 ± 27.0	12.773	<0.001^a^	14.5	12.3	16.8
Height (cm)	155.3 ± 23.9	154.5 ± 25.4	0.588	0.555	0.7	−1.7	3.1

a Statistically significant.

**Table 3:** Symptoms of malaria

Variable	Comorbidity (*n* = 979)		No Comorbidity (*n* = 999)		*χ* ^2^	*P* ‐value
	Frequency	Percent	Frequency	Percent		
Fever	590	60.3	462	46.2	39.030	<0.001^a^
Headache	645	66.0	401	40.1	131.510	<0.001^a^
Joint pain s	516	52.7	389	38.9	37.763	<0.001^a^
Abdominal pains	227	23.2	197	19.7	3.530	0.060
Vomiting	93	9.5	180	18.0	30.159	<0.001^a^
Diarrhea	54	5.5	83	8.3	5.981	0.014^a^
Nausea	166	17.0	148	14.8	1.698	0.193
Tiredness	390	39.8	498	49.8	20.040	<0.001^a^
Weakness	513	52.4	445	44.5	12.218	<0.001^a^
Dizziness	175	17.9	60	6.0	66.537	<0.001^a^
Body pains	526	53.7	318	31.8	96.910	<0.001^a^
Chills & Rigors	192	16.6	120	12.0	21.496	<0.001^a^
Bitter taste	380	38.8	116	11.6	194.758	<0.001^a^
Cough	252	25.7	277	27.7	0.997	0.318
Poor appetite	305	31.2	201	20.1	31.622	<0.001^a^
Temperature	145	14.8	120	12.0	3.339	0.068

a Statistically significant *df* = 1

Out of the 979 patients with comorbidity, 352 (36%) were hypertensive, 22 (2.2) were hypertensive‐diabetes, others were peptic ulcer disease 161 (16.4%), HIV/AIDS 102 (10.4%), Diabetes 43 (4.4%), Asthma 42 (4.3%), osteoarthritis 30 (3.1%), and sickle cell disease 29 (3.0%). Although pregnancy is a physiologic condition, it was classified under comorbid conditions because of the special considerations with the use of artemisinin combination therapy Table 4.

**Table 4:** Types and frequency of ADRs recorded

ADR	Patients with comorbidity *N* = 731 (%)	Patients without comorbidity *N* = 374(%)	Total *N* = 1105(%)
CNS			
Dizziness	86 (11.8)	67 (19.9)	153 (13.9)
Headaches	54 (7.3)	35 (9.4)	89 (8.1)
Insomnia	21 (2.9)	1 (0.3)	22 (2.0)
Drowsiness	14 (1.9)	3 (0.8)	17 (1.5)
Restlessness	5 (0.7)	7 (1.9)	12 (1.1)
Tiredness	6 (0.8)	4 (1.1)	10 (0.9)
Excessive sweating	7 (1.0)	1 (0.3)	8 (0.7)
Palpitations	3 (0.4)	4 (1.1)	7 (0.6)
Somnolence	3 (0.4)	3 (0.8)	6 (0.5)
Tremor	5 (0.7)	0 (0.0)	5 (0.5)
Blurred visions	3 (0.4)	1 (0.3)	4 (0.4)
Internal heat	0 (0.0)	3 (0.8)	3 (0.3)
Migraine	2 (0.3	0 (0.0)	2 (0.2)
Paraesthesiae	1 (0.1)	0 (0.0)	1 (0.1)
Tinnitus	1 (0.3)	0 (0.0)	1 (0.1)
Musculoskeletal			
Weakness	263 (36.0)	101 (27.0)	364 (32.9)
Low Back Pain	11 (1.5)	11 (2.9)	22 (2.0)
Back pain	9 (1.2)	7 (1.9)	16 (1.4)
Joint pains.	4 (0.6)	10 (2.7)	14 (1.3)
Fatigue	5 (0.7)	6 (1.6)	11 (1.0)
Myalgia	1 (0.1)	3 (0.8)	4 (0.4)
Jaw swellings	2 (0.3)	0 (0.0)	2 (0.2)
Neck swelling	1 (0.1)	0 (0.0)	1 (0.1)
Malaise	0 (0.0)	1 (0.3)	1 (0.1)
Gastrointestinal			
Vomiting	29 (4.0)	30 (8.0)	59 (5.3)
Nausea	36 (4.9)	14 (3.7)	50 (4.5)
Abdominal pain	13 (1.8)	16 (4.3)	29 (2.6)
Diarrhea	21 (2.9)	8 (2.1)	29 (2.6)
Increased appetite	13 (1.8)	6 (1.6)	19 (1.7)
Abdominal discomfort	5 (0.7)	5 (1.3)	10 (0.9)
Bitter taste	7 (1.0)	2 (0.5)	9 (0.8)
Loss of appetite	8 (1.1)	1 (0.3)	9 (0.8)
Anorexia	5 (0.7)	2 (0.5)	7 (0.7)
Epigastric pain	3 (0.4)	2 (0.5)	5 (0.5)
Abdominal cramps	0 (0.0)	2 (0.5)	2 (0.2)
Constipations	2 (0.3)	0 (0.0)	2 (0.2)
Cardiovascular			
Fainting Attack/collapse	3 (0.4)	1 (0.3)	4 (0.4)
Hypertension	1 (0.1)	0 (0.0)	1 (0.1)
Pedal swelling	1 (0.1)	0 (0.0)	1 (0.1)
Hot Flushes	1 (1.1)	0 (0.0)	1 (0.1)
Respiratory			
URTI	17 (2.3)	4 (1.1)	21 (2.0)
Cough	4 (0.6)	1 (0.3)	5 (0.5)
Chest pains	3 (0.4)	1 (0.3)	4 (0.4)
Difficulty in breathing	2 (0.3)	0 (0.0)	2 (0.2)
Heaviness in chest	0 (0.0)	1 (0.3)	1 (0.1)
Renal			
Hematuria	3 (0.4)	0 (0.0)	3 (0.3)
Yellowish urine	3 (0.4)	0 (0.0)	3 (0.3)
Increased frequency	2 (0.3)	0 (0.0)	2 (0.2)
Facial swelling	1 (0.1)	0 (0.0)	1 (0.1)
Dermatological			
Itching	8 (1.1)	1 (0.3)	9 (0.9)
Rashes	0 (0.0)	3 (0.8)	3 (0.3)
General			
Fever	25 (3.4)	7 (1.9)	32 (3.0)
Chills & rigors	3 (0.4)	1 (0.3)	4 (0.4)
Metabolic			
Excessive thirst	3 (0.4)	0 (0.0)	2 (0.2)
Jaw swellings	2 (0.3)	0 (0.0)	2 (0.2)
Hypoglycemia	1 (0.1)	0 (0.0)	1 (0.1)
Neck swelling	1 (0.1)	0 (0.0)	1 (0.1)

### Types and frequency of suspected adverse drug reactions

Table 4 presents the types and frequency of ADRs recorded. There were 1105 suspected adverse drug reactions in all, of which 731 (66.2%) were recorded in patients with comorbidity, and 374 (34%) were in patients without comorbidity. The most predominant symptoms are weakness 364, dizziness 153, headache 89, vomiting 59, nausea 50, fever 32, abdominal pain 29, and insomnia 22. There were 1105 suspected adverse drug reactions in all, of which 731 (66.2%) were recorded in patients with comorbidity, and 374 (34%) were in patients without comorbidity. The most predominant events seen among those with comorbidity includes weakness 263(36%), dizziness 86 (11.8%), headache 54 (7.3), nausea 36 (4.9%), vomiting 29 (4.0%) and insomnia 21 (2.9%). However, in those with comorbidity, the most predominant events seen includes body weakness 101 (27%), dizziness 67 (19.1%), headache 35 (9.4%), vomiting 30 (8.8%), abdominal pain 16 (4.3%), and nausea 14 (3.4%). This gives prevalence of 19.6% among patients with co‐morbidity, while that of patients without co‐morbidity was 7.6%. Patients with co‐morbidity were three times more likely to have adverse drug reaction than those without co‐morbidity and it could be as low as two times and as high as four times (Odds ration = 2.96; 95% CI = 2.23–3.93) Table 5.

**Table 5:** Occurrence of adverse drug reaction (ADR)

Co‐morbidity	Adverse drug reaction			Odds ratio	95% CI	
	Yes	No	Total		Lower	Upper
Yes	192 (19.6)	787 (80.4)	979	2.96^a^	2.23	3.93
No	76 (7.6)	923 (92.4)	999	1		
Total	268	1710	1978			
*χ* ^2^ = 60.829; *df* = 1; *P* < 0.001^a^						

a Statistically significant.

### Potential risk factors analysis

We subjected our finding to multinomial logistic regressions for potential risk factors and the presence of comorbidity and causation of ADR. The presence of comorbidity was found to be statistically significant with causation of AEs, *P *< 0.0001 (CI: 1.98–3.96), In relating to specific types of comorbidity and the risk of developing an adverse events, HIV/AIDS and osteoarthritis were significantly (*P *< 0.00) more associated with development of adverse events in this cohorts. Also age, weight and height of patients appears to be associated risk for adverse events *P *<* *0.0001 (CI: 1.02–1.04), *P* < 0.001 (CI: 1.017–1.03), and *P* < 0.001 (CI: 1.007–1.02), respectively Table 6.

**Table 6:** Simple and multiple logistic regression (multivariable analysis) of occurrence of adverse drug reaction (adr) on the associated factors

Variable	Simple logistic regression			*P* ‐value	Multiple logistic regression			*P* ‐value
	Crude Odds ratio	95% CI			Adjusted Odds ratio	95% CI		
		Lower	Upper			Lower	Upper	
Comorbidity (Yes/No)	2.80^a^	1.98	3.96	<0.001^a^	2.32^a^	1.48	3.65	<0.001^a^
Age (year)	1.03^a^	1.02	1.04	<0.001^a^	1.01	0.99	1.02	0.581
Sex (Female/Male)	1.19	0.84	1.68	0.322				
Weight (kg)	1.024^a^	1.017	1.03	<0.001^a^	1.01	0.99	1.02	0.394
Height (cm)	1.014^a^	1.007	1.02	<0.001^a^	1.01	0.992	1.02	0.288
Traditional Medication (Yes/No)	0.35^a^	0.23	0.52	<0.001^a^	0.35^a^	0.21	0.57	<0.001^a^
Types of Antimalarial received								
Artemether–Amodiaquine	0.854	0.1.453	3.798	<0.001^a^	0.245	0.638	1.014	<0.001^a^
Artemether–Lumefantrine	–	–	–	–	–	–	–	–
Pregnancy (Yes/No)		0.144	18.562	0.691	0.493	1.085	67.901	0.042

a Statistically significant.

## Discussion

Artemisinin‐based combination therapy (ACT) as “antimalarial drugs, is the simultaneous use of two or more blood schizontocidal drugs with independent modes of action and thus unrelated biochemical targets in the parasite” ( Rashidul et al. 2007). ACTs as a chemotherapeutic agents can accelerate therapeutic response, improve cure rates and protect the component drugs against resistance (Field et al. 2001). However, the widespread nature of malaria infections all over the sub‐Saharan African countries especially Nigeria has led to a situation where it is common for malaria patients to have concurrent co‐infection with other disease such that when one is treating malaria, there is background comorbid conditions requiring additional therapy. This scenario has led to administration of polypharmacy to address multiple diseases which in turn make malaria patients more vulnerable to ADRs as is often the case with patients who has presence of many diseases and the use of many drugs.

Patients with co‐morbidity were found to be three times more likely to have these adverse drug reaction in our studies than those without co‐morbidity and it could be as low as two times and as high as four times (Odds ration = 2.96; 95% CI: 2.23–3.93). Also age, weight, and height of patients appears to be associated risk for adverse events *P* < 0.0001 (CI: 1.02–1.04), *P* < 0.001 (CI: 1.017–1.03), and *P* < 0.001 (CI: 1.007–1.02). This is not surprising as risk factors reported to be independently associated with repeat admission due to ADRs included comorbidity, sex, multiple drug regimens and inappropriate use of medication, alcohol intake, poor cognitive function, and depression. (Carbonin et al. 1991; Field et al. 2001; Onder et al. 2002, 2003; Fialová et al. 2005; Klarin et al. 2005; Lima and Melo 2012). Although more patients had hypertension, peptic ulcer disease and diabetes, co‐morbidity with HIV/AIDS and osteoarthritis were the conditions that were statistically significant associated with development of ADR in our study. HIV increases the risk of malaria infection and clinical malaria in adults, especially in those with advanced immunosuppression (Whitworth et al. 2000; French et al. 2001; Ned et al. 2005; Chalwe et al. 2009 ). In our study, the most common ADR recorded were weakness, dizziness, headache, vomiting, nausea, fever, abdominal pain, and insomnia which are in keeping with previous studies. (Linda et al. 2011; Bassi et al. 2012; Dodoo et al. 2014; WHO 2015). These symptoms were generally worse from the second and third day of treatment, but generally by the fifth day of drug administrations. In the settings with unstable malaria transmission, HIV‐infected adults are found to be at increased risk of complicated and severe malaria and (Table 7) death (Whitworth et al.^2000^; French et al. 2001; WHO 2015). Reports also suggest that antimalarial treatment failure may be more common in HIV‐infected adults with low CD4‐cell counts compared to those not infected with HIV (Whitworth et al.^2000^; French et al. 2001; Ned et al. 2005; Chalwe et al. 2009). The same pattern may apply to HIV and ADR due to ACT adverse drug reactions. Additional research is needed to investigate the impact of malaria on the natural history of HIV, potential therapeutic implications, and interactions at a cellular and molecular level. Furthermore, when AA was prescribed to HIV/AIDs patients on some anti‐retroviral drugs, ADRs were found to have increased (German et al. 2007). For example Efervirenz (EFV) is a potent inhibitor of CYP2C8 enzyme in vitro and may, therefore, potentially increase the plasma levels of amodiaquine when coadministered (German et al. 2007). However, the interaction is not expected to affect the therapeutic efficacy of the antimalarial, as both amodiaquine and its metabolite, N‐desethylamodiaquine, are active antimalarials, but it may have implications for toxicity. German et al. (2007) and Churchill et al. (1985) reported a case of 1.5–3fold increase in the area under the plasma concentration curve for amodiaquine when coadministered with EFV and artesunate to two HIV‐infected patients. The interaction between the drugs also resulted in asymptomatic but significant elevations in hepatic transaminases. Uriel and Lewthwaite (2011) also reported a case of an HIV‐infected patient on abacavir (ABC), lamivudine (3TC), and NVP who also had *Plasmodium falciparum* malaria. More studies are therefore needed to explore the effects of AA coo administration in these medicines and as they are key first line drugs in HIV management in Nigeria.

**Table 7:** Association between type of comorbidity and adverse drug reaction (ADR)

Type of comorbidity	Adverse drug reaction			*χ* ^2^	*P*‐value
	Yes (*n* = 192)	No (*n* = 787)	Total		
Hypertension	81 (42.2)	271 (77.0)	352	4.093	0.045
Peptic ulcer disease	28 (17.4)	133 (82.6)	161	0.603	0.438
Human immunodeficiency virus	8 (7.8)	94 (92.2)	102	10.003	0.002^a^
Diabetes mellitus	6 (14.0)	37 (86.0)	43	0.913	0.339
Asthma	6 (14.3)	36 (85.7)	42	0.790	0.374
Osteoarthritis	13 (43.3)	17 (56.7)	30	11.046	0.001^a^
Sickle cell disease	3 (10.3)	26 (89.7)	29	1.628	0.202
Hypertension/diabetes mellitus	5 (22.7)	17 (77.3)	22	0.139	0.710
Pregnancy	4 (28.6)	10 (71.4)	14	0.723	0.395
Low back pain	2 (15.4)	11 (84.6)	13	0.149	0.699
Pelvic inflammatory disease	1 (8.3)	11 (91.7)	12	0.980	0.322
Tuberculosis	3 (25.0)	9 (75.0)	12	0.224	0.636
Difficulty in seeing	5 (41.7)	7 (58.3)	12	3.748	0.053
Injury	3 (27.3)	8 (72.7)	11	0.414	0.520
Insomnia	0 (0)	9 (100)	9	2.216	0.137
Seizures	1 (11.1)	8 (88.9)	9	0.416	0.519
Surgery	2 (22.2)	7 (77.8)	9	0.039	0.843
Others	21 (21.6)	76 (78.4)	97	0.284	0.594

a Statistically significant.

Osteoarthritis has been consistently associated with factors that predict ADRs. Recently, Zhang et al. 2009 observed in a retrospective cohort study of comorbidity and repeat admission to hospital for adverse drug reactions in older adults, that Compared with patients who had no recorded comorbidity, the analysis identified sizeable adjusted hazard ratios that comorbidity with rheumatological disease, congestive cardiac failure, peripheral vascular, chronic pulmonary, hepatic, renal and malignant diseases predict readmission for ADRs (Zhang et al. 2009).

Regardless of symptoms, the presence of plasmodial parasites in a pregnant woman's body will have a negative impact on her own health and that or her fetus. Malaria in pregnancy is different from the disease in the non‐pregnant state. The increased severity of malaria in pregnancy is thought to be due to general impaired immunity plus a diminution of acquired immunity to malaria in endemic areas. Placental malaria occurs where *Plasmodium falciparum*‐infected erythrocytes accumulate in the intervillous space of the placenta but may be rare or absent in the peripheral circulation (Chalwe et al. 2009; Bassi et al. 2012; Dodoo et al. 2014). One would expect pregnancy to be significantly associated with ADR going by previous findings by Bassi et al. 2012; but this was not the case in this study. This is probably because of the insufficient number of pregnant patients 14 (1.4%) seen in the cohort. Preventing and treating malaria in pregnancy can be a key intervention to improving maternal, fetal and child health globally and are linked to three of the Millennium Development Goals (UN. Report 2015).

Taking AA is significantly associated with ADR in our studies. This is in keeping with findings in literature (McGready et al. 2001; Bassi et al. 2012; Chatio et al. 2016). Though no pharmacokinetic interactions have been documented, amodiaquine and desethylamodiaquine inhibit CYP 2D6 in vitro and may cause clinically significant interactions with some *β*‐blockers, antidepressants, and antipsychotics drugs. The presence of many patients with hypertension might have allow drug–drug interactions. It therefore underscore the importance of cautions while treating patients with malaria and comorbid conditions to pay special attention to drug–drug interactions and pharmacovigilance.

## Conclusions

Patients with co‐morbidity were three times more likely to have adverse drug reaction in our studies than those without co‐morbidity and it could be as low as two times and as high as four times. Although more patients had hypertension, peptic ulcer disease and diabetes, co‐morbidity with HIV/AIDS and osteoarthritis were the conditions that were statistically significant in causing ADR in our study. It is therefore recommended that clinicians should ensure close monitoring of patients with comorbid conditions to optimize therapy and prevent or minimized AEs as we call for more studies in these areas.

## Author's Contribution

Peter U. Bassi, Osakwe AO (now retired), and Suku Comfort, were responsible for this study and designed the proposal and oversaw the work. B, UP was responsible for database extraction, data entry, and pre‐analysis management. Casandra Elegbaje, Sulayman T. Balogun and Godwin N. Ntadom were the research associates implementing the study, managing the data collections during the initial program. Biyaya, BNwankwo performed the statistical analysis. Peter U. Bassi drafted the manuscript, Ambrose, O Isah revised it critically for important intellectual content and all authors read and approved the final manuscript

## Conflict Of Interest

No competing interest to declare. The opinions expressed in this paper are those of the authors and may not reflect those of their employing organizations. Dr Peter U. Bassi is a staff member of the University of Abuja, Nigeria; Osakwe AO (now retired), Suku Comfort, Casandra Elegbaje are staff member of NPC‐NAFDAC, Nigeria; the authors alone are responsible for the views expressed in this publication and they do not necessarily represent the decisions, policy or views of the University of Abuja, NAFDAC Nigeria or Government of Nigeria.
